# Macromolecular structure determination using X-rays, neutrons and electrons: recent developments in *Phenix*

**DOI:** 10.1107/S2059798319011471

**Published:** 2019-10-02

**Authors:** Dorothee Liebschner, Pavel V. Afonine, Matthew L. Baker, Gábor Bunkóczi, Vincent B. Chen, Tristan I. Croll, Bradley Hintze, Li-Wei Hung, Swati Jain, Airlie J. McCoy, Nigel W. Moriarty, Robert D. Oeffner, Billy K. Poon, Michael G. Prisant, Randy J. Read, Jane S. Richardson, David C. Richardson, Massimo D. Sammito, Oleg V. Sobolev, Duncan H. Stockwell, Thomas C. Terwilliger, Alexandre G. Urzhumtsev, Lizbeth L. Videau, Christopher J. Williams, Paul D. Adams

**Affiliations:** a Molecular Biophysics and Integrated Bioimaging Division, Lawrence Berkeley National Laboratory, Berkeley, CA 94720, USA; b Verna and Marrs McLean Department of Biochemistry and Molecular Biology, Baylor College of Medicine, Houston, TX 77030, USA; c Department of Haematology, Cambridge Institute for Medical Research, University of Cambridge, Cambridge CB2 0XY, England; d Department of Biochemistry, Duke University, Durham, NC 27710, USA; e Los Alamos National Laboratory, Los Alamos, NM 87545, USA; f New Mexico Consortium, Los Alamos, NM 87544, USA; g Centre for Integrative Biology, Institut de Génétique et de Biologie Moléculaire et Cellulaire, CNRS–INSERM–UdS, 67404 Illkirch, France; h Faculté des Sciences et Technologies, Université de Lorraine, BP 239, 54506 Vandoeuvre-lès-Nancy, France; i Department of Bioengineering, University of California Berkeley, Berkeley, CA 94720, USA

**Keywords:** *Phenix*, automation, macromolecular crystallography, cryo-EM, X-rays, neutrons, diffraction, Python, *cctbx*, C++

## Abstract

Diffraction (X-ray, neutron and electron) and electron cryo-microscopy are powerful methods to determine three-dimensional macromolecular structures, which are required to understand biological processes and to develop new therapeutics against diseases. The overall structure-solution workflow is similar for these techniques, but nuances exist because the properties of the reduced experimental data are different. Software tools for structure determination should therefore be tailored for each method. *Phenix* is a comprehensive software package for macromolecular structure determination that handles data from any of these techniques. Tasks performed with *Phenix* include data-quality assessment, map improvement, model building, the validation/rebuilding/refinement cycle and deposition. Each tool caters to the type of experimental data. The design of *Phenix* emphasizes the automation of procedures, where possible, to minimize repetitive and time-consuming manual tasks, while default parameters are chosen to encourage best practice. A graphical user interface provides access to many command-line features of *Phenix* and streamlines the transition between programs, project tracking and re-running of previous tasks.

## Abstract 2

Recent developments in the *Phenix* software package are described in the context of macromolecular structure determination using X-rays, neutrons and electrons.

## 1. Introduction   

**Figure 1:**
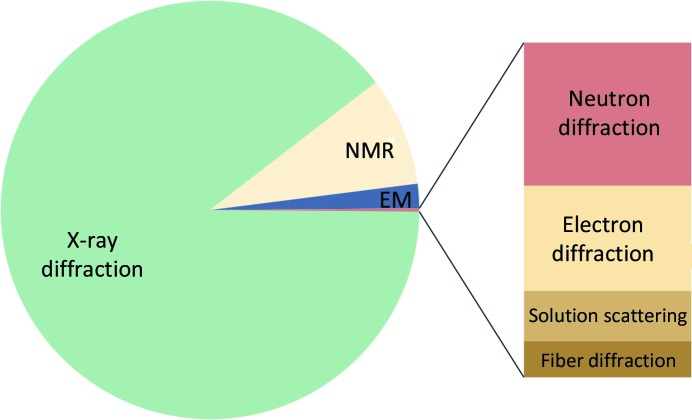
Experimental methods used to determine macromolecular structures that are deposited in the PDB. The predominant method is X-ray diffraction, followed by nuclear magnetic resonance (NMR), cryo-EM and neutron diffraction.

Macromolecules are essential for biological processes within organisms, engendering the need to understand their behavior to explain the fundamentals of life. The function of macromolecules correlates with their three-dimensional structure, *i.e.* how the atoms of the molecule are arranged in space and how they move over time. Two major methods to obtain macromolecular structures are diffraction (usually using X-rays, but also neutrons or electrons) and electron cryo-microscopy (cryo-EM^1^) (Fig. 1 ▸), both of which are handled by *Phenix*. The following subsections describe some concepts underpinning each method for the benefit of readers who are not experts in each of these areas.

### 1.1. X-ray diffraction   

X-ray diffraction relies on the interaction of X-rays with the electron cloud of atoms in a crystal. As the atomic core electron density dominates the electron-density distribution, major peaks equate to atomic positions and can be used to determine the structure. An exception is the H atom because it possesses only one valence electron, the distribution of which is shifted towards its covalent-bond partner. The electron density in the unit cell is related to the Fourier transform of the amplitude and phase of the scattered X-rays. As only the intensities of the waves can be measured, the phase information is lost and has to be inferred by various methods (Section 4.1).

Of the models deposited in the Protein Data Bank (PDB; Bernstein *et al.*, 1977 ▸; Burley *et al.*, 2019 ▸), 89% originate from X-ray crystallography. Since the first protein structures were determined in the 1950s (Kendrew *et al.*, 1958 ▸; Perutz *et al.*, 1960 ▸), the method has experienced many methodological and technological developments and is now considered to be quite mature (Wlodawer *et al.*, 2013 ▸). Nevertheless, structures determined at low resolution (for example, worse than 3 Å) remain challenging and could benefit from some of the new developments for cryo-EM that target similar resolution ranges.

### 1.2. Neutron diffraction   

Neutron diffraction, which relies on the same formalism as X-ray diffraction, is based on the interaction of neutrons with atomic nuclei and therefore yields actual atomic positions directly. As the neutron scattering cross-section varies by element (or isotope) in a nonlinear fashion, the scattering lengths of light atoms such as hydrogen and deuterium (D) atoms are similar to those of the heavier atoms (C, O and N). It is therefore possible to locate H (or D) atoms and to deduce their protonation states; this knowledge helps in understanding catalytic mechanisms and ligand binding (Yamaguchi *et al.*, 2009 ▸; Bryan *et al.*, 2013 ▸; Knihtila *et al.*, 2015 ▸). Furthermore, the neutron scattering length can be positive or negative (it is always positive for X-rays). For example, H has a negative scattering length, the magnitude of which is about half of the scattering length of carbon. The nuclear scattering length density can therefore cancel out for groups such as CH_2_, which occur frequently in macromolecules. To avoid negative scattering from H atoms, hydrogen can be partially or fully exchanged with deuterium by soaking the crystal in deuterated buffer solutions or by performing protein expression in fully deuterated reagents, respectively.

The number of structures determined by neutron crystallography (0.1% of the models deposited in the PDB) is small compared with the number of X-ray structures (89%). Neutron diffraction is not used to solve the structure of a macromolecule *de novo* as it requires considerable effort to prepare deuterated crystals suitable for the experiment. Instead, neutron diffraction provides complementary information because it enables the location of H or D atoms.

### 1.3. Cryo-EM   

Cryo-EM relies on the interaction of electrons with the electrostatic field of the atoms in the sample. The method comprises many techniques, such as electron tomography, electron single-particle microscopy and electron crystallo­graphy. Single-particle analysis is a commonly used variant that combines 2D projection images of macromolecules into a 3D reconstruction (electrostatic potential map or cryo-EM map). This is in contrast to diffraction experiments, in which the phase information is lost (in the absence of experimental phases, electron-density maps thus have to be calculated using a model). While being visually similar to electron-density maps from X-ray diffraction, a cryo-EM map exhibits some differences, such as negative peaks from negatively charged nucleic acids (Wang & Moore, 2017 ▸). Furthermore, the reconstruction process and motion or heterogeneity of the sample can lead to blurring of cryo-EM maps; high-resolution details can be revealed by operations such as map sharpening (Section 4.2).

**Figure 2:**
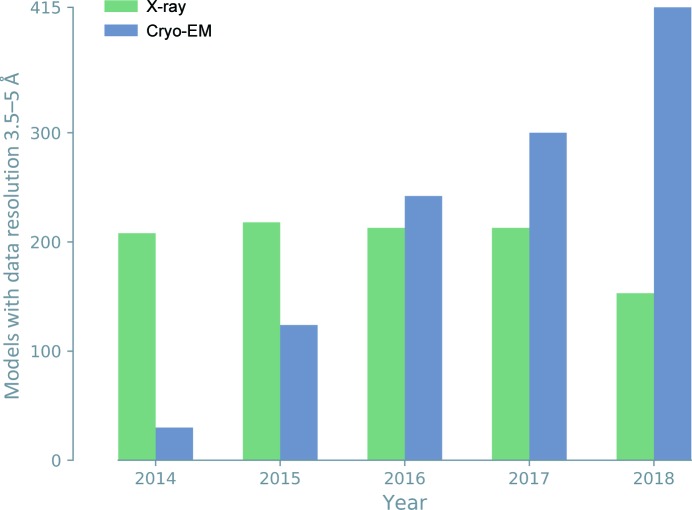
Annual cryo-EM model depositions now outnumber X-ray model depositions in the resolution range 3.5–5 Å.

Cryo-EM was traditionally employed to investigate large protein and nucleic acid complexes, filaments and viruses, but was often limited to resolutions worse than 5 Å. Technological advances, such as the development of direct electron detectors (Li *et al.*, 2013 ▸) and improvements in image processing (Bai *et al.*, 2015 ▸), have transformed the method, leading to cryo-EM maps with greatly improved resolution. More recently, 3D reconstructions have routinely attained resolutions significantly better than 4 Å, allowing atomic model interpretation and the solution of structures *de novo*. Cryo-EM has thus become another principal method of macromolecular structure determination (2% of the models deposited in the PDB). For large molecules and structures determined at low resolution, annual depositions of cryo-EM models now outnumber those of X-ray models (Figs. 2 ▸ and 3 ▸).

### 1.4. Other techniques   

Another method to determine macromolecular structures is NMR (nuclear magnetic resonance; 9% of the models deposited in the PDB), which uses the quantum properties of atomic nuclei. *Phenix* does not have tools for structure determination using NMR data, so it is not addressed here.

Electron diffraction on nearly single-layer crystals is an emerging technique to determine high-resolution structures of macromolecules. It accounts for a slightly smaller number of models in the PDB than neutron diffraction.

### 1.5. *Phenix*   

*Phenix* (Adams *et al.*, 2002 ▸, 2010 ▸) is a software suite that uses reduced data from X-ray diffraction, electron diffraction, neutron diffraction or cryo-EM to determine macromolecular structures. Each method has a different approach to derive structural information, with *Phenix* offering specific tools to address the unique properties of the experimental data. Emphasis is put on the automation of all procedures to avoid burdening the user with repetitive, time-consuming and often error-prone tasks. Another important feature is the user-friendly design, which makes the program accessible to novice users while keeping it flexible for experts. New tools are regularly developed or enhanced to improve the structure-solution workflow. For example, a series of programs which focuses on the analysis of cryo-EM maps and models has recently been created to answer the emerging needs of the cryo-EM community.

This article describes the structure-determination process of three methods (X-ray diffraction, neutron diffraction and cryo-EM), summarizes major tools and reports recent develop­ments in *Phenix*.

## 2. Steps in the structure-solution process   

**Figure 4:**
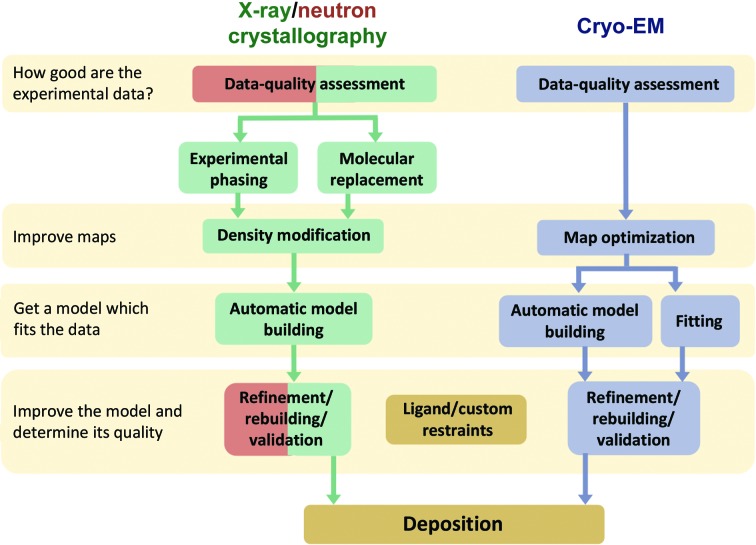
The structure-solution steps for X-ray/neutron crystallography and cryo-EM have nuances for each technique, but the overall workflow is similar. Color code: cryo-EM, gray; X-ray crystallography, green; neutron crystallography, red. As neutron diffraction experiments are typically performed with samples for which the structure is known, the phasing, density modification and model-building steps are not part of the workflow.

**Figure 5:**
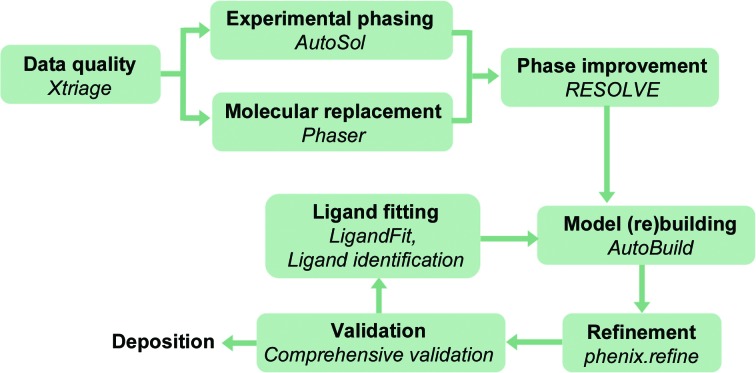
The primary tools for X-ray crystallography in *Phenix*.

Fig. 4 ▸ shows the steps of the structure-solution process for X-ray/neutron crystallography and cryo-EM. Owing to the different nature of the interactions, there are nuances for each structure-determination method (Figs. 5 ▸ and 6 ▸), but the overall procedure to obtain a molecular model is similar. The first step consists of analyzing the derived experimental data to detect any anomalies that can complicate or even prevent structure determination (Section 3). The second and third steps are to obtain the best possible map (Section 4) so that a model can be built (Section 5). The fourth step focuses on iteratively improving the model by cycles of local rebuilding, refinement and validation (Sections 6 and 7). The subsequent sections elaborate on the steps and explain similarities and differences for X-ray/neutron crystallographic and cryo-EM data. The *Phenix* tools that perform the corresponding steps are described.

## 3. Data-quality assessment   

Data quality should be analyzed carefully because unusual features can thwart structure solution. If the data have anomalies, it is not guaranteed that they can be addressed at later stages, in which case it might be necessary to perform new experiments or re-analyze the raw data.

### 3.1. Crystallography   

#### 3.1.1. *Xtriage*   

Macromolecular crystals are prone to pathologies and rarely achieve perfect order, as the molecules interact weakly with each other. For example, a crystal is called ‘twinned’ if two or more crystals (domains) are intergrown in such a way that their orientations are related by a specific geometrical operation (a twin operation; Hahn & Klapper, 2006 ▸). The overlap of diffraction spots adds noise to the measurements and reduces the information content of the data. Translational noncrystallographic symmetry (tNCS) is another pathology that complicates structure determination. This arises when more than one copy of a molecule or assembly is found in a similar orientation in the asymmetric unit of the crystal. Interference effects between diffraction from the copies lead to an overall modulation of the intensities in the diffraction pattern.

The program *Xtriage* (*phenix.xtriage*) identifies twinning, tNCS and other unusual features of diffraction data (Zwart *et al.*, 2005 ▸). To detect twinning, the tool examines amplitude and intensity ratios, |*E* ^2^ − 1| values, the *L*-statistic (Padilla & Yeates, 2003 ▸) and *N*(*Z*) plots (Howells *et al.*, 1950 ▸). The twin fraction is estimated by interpreting the Britton plot^2^ (Britton, 1972 ▸) and performing the *H*-test (Yeates, 1997 ▸). *Xtriage* reveals tNCS using the native Patterson function and uses database-derived Wilson plots to find anomalies in the mean intensity. The tool also analyses reflection merging statistics to detect whether the input data symmetry is too low, and systematic absences to identify screw axes. A warning is issued if ice rings are detected. Apart from identifying pathologies, *Xtriage* also reports data-quality indicators, such as the signal-to-noise ratio and data completeness. Furthermore, the tool estimates anomalous signal strength based on the fraction of statistically significant Bijvoet differences (Zwart, 2005 ▸) and the overall anisotropic scale factor using the likelihood formalism described by Popov & Bourenkov (2003 ▸).

#### 3.1.2. Planning and assessing a SAD experiment   

Before conducting a single-wavelength anomalous diffraction (SAD) experiment, it is useful to assess its chances of success. *Plan SAD experiment* (*phenix.plan_sad_experiment*) is a new tool for estimating the anomalous signal from a SAD experiment with a particular anomalous scatterer and data quality, and for predicting whether this signal would be sufficient to solve the structure (Terwilliger *et al.*, 2016*a* ▸,*b* ▸). The tool provides a summary of the anomalous signal required and what can be expected if the data can be measured with the suggested overall signal-to-noise ratio (*I*/σ). Once data have been collected and then scaled with the *Scale and Merge Data* (*phenix.scale_and_merge*) tool, the *Anomalous Signal* (*phenix.anomalous_signal*) tool estimates the amount of signal that has actually been achieved and predicts whether or not this will be sufficient to solve the structure.

### 3.2. Cryo-EM: *Mtriage*   

The sample for a cryo-EM experiment is not crystalline, so many of the problems discussed in the previous section (Section 3.1.1) are not relevant. However, the quality of the reconstruction and therefore the interpretability of a cryo-EM map can deteriorate from many causes, such as structural heterogeneity, radiation damage and beam-induced sample movement. The information content of a cryo-EM map is typically expressed by the resolution. While the same term (‘resolution’) is used in crystallography to describe data quality, its meaning differs: the resolution of a crystallo­graphic data set depends on the largest angle to which diffracted beams were measured or, equivalently, the shortest distance between reciprocal-lattice planes (McPherson, 2009 ▸). The overall resolution *d*_FSC_ of a cryo-EM map is usually defined as the maximum spatial frequency at which the information content of the map is reliable (Penczek, 2010 ▸). The value is obtained by analyzing the Fourier shell correlation (FSC) for two cryo-EM half-maps binned in resolution shells (van Heel & Harauz, 1986 ▸). If the macromolecule is structurally heterogeneous (for example flexible regions in the macromolecule), a single value for the resolution is most likely to be inadequate. A ‘local resolution’ is thus assigned to different map regions (Cardone *et al.*, 2013 ▸; Kucukelbir *et al.*, 2014 ▸).

In *Phenix*, the resolution of cryo-EM maps can be estimated with the newly developed tool *Mtriage* (*phenix.mtriage*; Afonine, Klaholz *et al.*, 2018 ▸) using several different approaches, some of which fundamentally differ from *d*_FSC_. The tool also summarizes map statistics. As the map resolution strongly influences the decisions made in subsequent steps, it is important to obtain a reliable estimate.

### 3.3. Common map tools   

Several new tools are available to analyze cryo-EM maps (or any map). In the context of molecular densities, a map is a 3D grid of density values. The map has an origin and a gridding (the distance between neighboring grid points). A map typically extends only over grid points where the values are nonzero (and a buffer), but it is possible that a majority of map points are zero or very small. Especially for cryo-EM, the molecules can have symmetry (such as viruses) and the map will have the same symmetry. The following tools analyze maps and perform some basic operations.

(i) *Show map info* (*phenix.show_map_info*) lists the properties of a map, such as origin, grid points, unit cell and map size.
(ii) *Map box* (*phenix.map_box*) cuts out a box from a large map.
(iii) Some molecules, such as viruses, can have high internal symmetry. It can thus be beneficial to reduce the map to the repeating unit. *Map symmetry* (*phenix.symmetry_from_map*) finds such symmetries and *Map box* can extract the unique part of the map.
(iv) The tool *Combine focused maps* (*phenix.combine_focused_maps*) creates a weighted composite map from a set of locally focused maps and associated models, where each part of each map is weighted by its correlation with the corresponding model.

## 4. Optimizing maps   

### 4.1. X-ray   

To calculate maps, the phase information is required. As phases are lost in the diffraction experiment, they have to be recovered by additional experiments or by computational procedures. In *Phenix*, phases can be determined by experimental phasing or by molecular replacement (Adams, Afonine *et al.*, 2009 ▸). Once an initial set of phases is known, they can be improved by optimizing electron-density maps or by optimizing an atomic model from which phases are calculated.

#### 4.1.1. Experimental phasing   

Experimental phasing relies on the properties of a few special atoms in the macromolecule. The special properties can be a large number of electrons, anomalous scattering, or a combination of both (reviewed by Dauter & Dauter, 2017 ▸). Phasing is then performed in two steps: (i) the properties are exploited to determine the location of the special atoms (the substructure) and (ii) knowledge of the substructure in one or more crystals is used to deduce phase information for the entire macromolecule.

*AutoSol* (*phenix.autosol*) is a comprehensive, automatic tool that performs experimental phasing with the MAD, MIR, SIR or SAD methods^3^ (Terwilliger *et al.*, 2009 ▸). The program locates the substructure, estimates phases, performs density modification, identifies noncrystallographic symmetry (NCS), and builds and refines a preliminary model. To carry out the tasks, *AutoSol* uses the *Phenix* tools *HySS* (*Hybrid Substructure Search*; Grosse-Kunstleve & Adams, 2003 ▸; Bunkóczi *et al.*, 2013 ▸), *SOLVE* (Terwilliger & Berendzen, 1999 ▸), *Phaser* (McCoy *et al.*, 2007 ▸), *RESOLVE* (Terwilliger, 2002 ▸), *Xtriage* and *phenix.refine* (Afonine *et al.*, 2012 ▸).

#### 4.1.2. Molecular replacement   

Molecular replacement (MR) is used to solve structures when a structurally similar model (homologue) is available (Hoppe, 1957 ▸; Rossmann, 1972 ▸; Blow *et al.*, 2012 ▸). Success in MR calculations is determined by how much signal can be extracted from the data using a particular model. This depends on a combination of the model quality and completeness, the resolution of the data and the number of diffraction observations (Oeffner *et al.*, 2018 ▸). For typical cases involving crystals of medium-sized proteins diffracting to moderate resolution, the sequence identity between the molecule and the homologue should be greater than 25–30% and the r.m.s. deviation between C^α^ atoms should be less than 2.0 Å (Taylor, 2010 ▸). The search model can be enhanced by trimming off parts of the model that are unlikely to be preserved in the target structure using *Sculptor* (*phenix.sculptor*; Bunkóczi & Read, 2011 ▸). The MR method consists of determining the orientation and position that places each copy of the homologue in the unit cell containing the unknown structure, judged by matching the calculated structure factors to the observed structure factors. An initial electron-density map is then calculated with the phases from the homologue and the observed structure factors (Evans & McCoy, 2008 ▸).

*Phaser* (*phenix.phaser*) applies maximum-likelihood principles (Bayesian probabilities) to crystal structure solution by MR, by single-wavelength anomalous diffraction (SAD), or by a combination of both (MR-SAD) (McCoy *et al.*, 2007 ▸). In common with most MR algorithms, it divides the six-dimensional search problem for each copy into a three-dimensional rotation search followed by a three-dimensional translation search. The use of maximum likelihood accounts for the effects of model imperfections and measurement error in the diffraction observations. In addition, likelihood provides a framework for the use of ensemble models created with *Ensembler* (*phenix.ensembler*) and for exploiting the placement of one copy to increase the signal of the search for another copy (McCoy *et al.*, 2007 ▸).

#### 4.1.3. Density modification   

As the initial phases are often quite inaccurate, they need to be improved by exploiting prior knowledge about electron-density distributions in crystals (Podjarny *et al.*, 1996 ▸). Examples of methods to improve phases are solvent flattening, histogram matching and, if NCS is present, noncrystallographic symmetry averaging.

Several *Phenix* programs carry out density modification. *phenix.density_modification* performs iterative phase improvement with *RESOLVE* (Terwilliger, 2000 ▸), including the use of NCS and electron-density distributions. *phenix.multi_crystal_average* improves phases iteratively and averages electron density, both within a crystal and between crystals. *Phenix.ncs_average* can be used to average the electron density for molecules related by NCS.

### 4.2. Cryo-EM: map optimization   

In the crystallographic case, map improvement is achieved by the manipulation of phase information, with the diffraction intensities (or amplitudes) remaining unchanged.^4^ In contrast, cryo-EM maps are improved by methods such as sharpening and blurring that typically modify the amplitudes of Fourier coefficients, leaving the phases unchanged. Cryo-EM maps can appear smooth and lack a high level of detail (contrast) because high-resolution amplitudes of corresponding Fourier map coefficients decay from causes such as radiation damage, sample movement, sample heterogeneity and errors in the reconstruction procedure. However, sharpening can reveal the high-resolution details concealed in a cryo-EM map (Rosenthal & Henderson, 2003 ▸; Fernández *et al.*, 2008 ▸).

The recently developed program *Autosharpen map* (*phenix.auto_sharpen*) performs map sharpening by optimizing the detail and connectivity of a cryo-EM map (Terwilliger, Sobolev *et al.*, 2018 ▸).

## 5. Obtaining a model that fits the experimental data   

**Figure 7:**
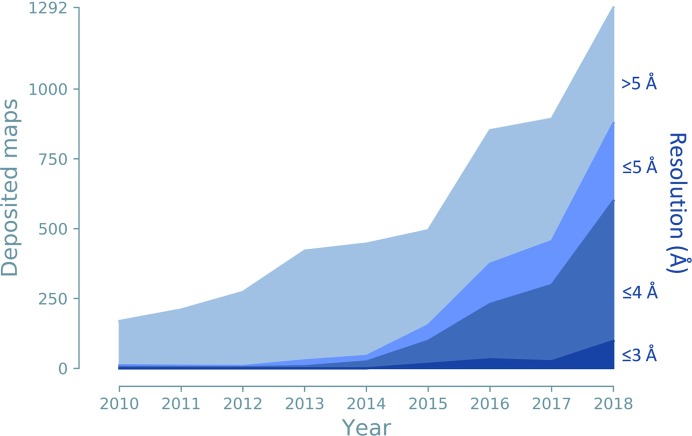
Cryo-EM maps deposited per year for different resolution ranges: better than 3 Å, 3–4 Å, 4–5 Å and worse than 5 Å.

To determine the structure of a macromolecule, a model must be built that fits the experimental data. Cryo-EM maps contain phase information, but they often have low resolution (Fig. 7 ▸), which makes interpretation difficult. For both techniques, but especially in the case of cryo-EM, the molecules can be very large, so that automated procedures are preferred over manual interpretation wherever feasible.

### 5.1. X-ray   

Automatic model building is performed after phasing because the models from MR or *AutoSol* might be too incomplete to carry out refinement immediately. MR models typically originate from a homologue model which has a different sequence and side-chain conformations; it is thus necessary to build a model according to the sequence of the target molecule. *AutoSol* includes a building step, but in order to optimize runtime it creates a preliminary model that can be further improved.

*AutoBuild* (*phenix.autobuild*) is an automated system for model rebuilding and completion (Terwilliger *et al.*, 2008 ▸). *AutoBuild* uses *RESOLVE*, *Xtriage* and *phenix.refine* to build an atomic model, refine it and improve it with iterative density modification, refinement and model building.

### 5.2. Cryo-EM   

To obtain a model that fits the cryo-EM map, the following procedures are available.

#### 5.2.1. Docking   

A docking procedure is used if the model or a part of the model is already known (Roseman, 2000 ▸) but has not yet been placed into the map. For example, the cryo-EM map might show a molecular complex assembled from components available from other experiments (such as crystallo­graphy). These components are then docked into the cryo-EM map to obtain a model for the entire complex.

The new tool *Dock in map* (*phenix.dock_in_map*) docks one or several models into a map (Terwilliger, 2018 ▸). The routine uses a convolution-based shape search to find a part of a map that is similar to the model. The shape search applies the following key elements. An initial search, focusing on the overall shape of the molecule, is performed at lower resolution. This initial search is performed without rotation to optimize runtime; it can optionally be supplemented by matching the moments of inertia of the model and map. If the placement is satisfactory, it is optimized using real-space rigid-body refinement with the full resolution of the map.

#### 5.2.2. Model building   

If the structure of the molecule or of its components is unknown, the model has to be built *ab initio* into the cryo-EM map. This task is challenging because the molecules are typically very large, chain tracing is difficult at low resolution and the effective resolution can be even lower in some regions. Manual interpretation of cryo-EM maps is therefore time-consuming and error-prone, so automatic procedures are desirable.

The recent tool *Map to model* (*phenix.map_to_model*) interprets a cryo-EM map and builds an atomic model (Terwilliger, Adams *et al.*, 2018 ▸). All steps are performed automatically. Firstly, the map is sharpened with *Autosharpen map* (Section 4.2). The unique parts of the structure are then identified by taking the reconstruction symmetry into account. The procedure also identifies which parts of the map correspond to protein or RNA/DNA. After atomic models have been generated, they are real-space refined using secondary-structure restraints (Section 6.1.3). To obtain optimal building results, the resolution of the map should be 4.5 Å or better.

## 6. Refinement   

Models from the building or phasing steps are approximate and need to be improved; for example, side chains may not fit the density, and water molecules and ligands are likely to be missing. Refinement is the process of improving the parameters of a model until the best fit is achieved between experimental and model-calculated data. The parameterization of an atomic model mainly depends on the data quality and the current stage of refinement. Generally, the parameterization is chosen such that a simpler model is used at the beginning (such as rigid body) and a more complex model is used towards the end. The target function guides the refinement by linking the model parameters to the experimental data and by scoring model-versus-data fit. For reciprocal-space refinement, the target function (*T*) is expressed through structure factors (or diffraction intensities). For real-space refinement, the target is formulated in terms of a map. In both cases, the process alternates automated refinement with valid­ation and either manual or automated model corrections.

### 6.1. Restraints   

Crystallographic and cryo-EM refinements need additional information because there are generally too many model parameters compared with the amount of experimental data (unless the resolution is better than ∼1 Å). Restraints introduce information and modify the target function by creating relationships between independent parameters. Using the example of restrained bond lengths, the coordinates of the two atoms are independent while the restraint keeps their distance within a certain target value and imposes a penalty if it deviates too much. Other restraints are imposed typically on bond angles, dihedrals, planes, chirality and coupling of atomic displacement parameters (ADPs) between bonded or neighboring atoms (Evans, 2007 ▸).

If restraints are used, the target function is a sum of an experimental-data component (*T*_data_) and a weighted restraints-based component (*w*_restraints_ × *T*_restraints_),
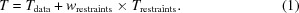



#### 6.1.1. Stereochemical restraints   

Proteins and nucleic acids (RNA and DNA) are composed of amino acids and nucleotides, respectively. The structures of these components are known from small-molecule crystallography, with the assumption that they are similar when they assemble to form a macromolecule. For bond lengths and angles, *Phenix* makes use of the CCP4 monomer library restraints (Engh & Huber, 1991 ▸; Vagin & Murshudov, 2004 ▸; Vagin *et al.*, 2004 ▸) in protein side chains and somewhat modified classic values for nucleic acids (Clowney *et al.*, 1996 ▸; Gelbin *et al.*, 1996 ▸). Planarity, dihedral angles and chirality are also restrained. Recent additions to the restraints used in *Phenix* include the conformation-dependent library (Berkholz *et al.*, 2009 ▸; Moriarty *et al.*, 2014 ▸, 2016 ▸), which restrains the protein main chain as a function of the backbone dihedral values. Ribose-pucker and base-type-dependent dihedral restraints are available for RNA (Jain *et al.*, 2015 ▸). Algorithms, as opposed to libraries, are also used to provide stereochemical restraints. Linking, including metal and metal cluster coordination (Moriarty & Adams, 2019 ▸), covalent bonding of standard and nonstandard carbohydrates and other specialized entity specific restraints can be performed automatically.

#### 6.1.2. Ligand restraints   

Ligands are small molecules that are bound covalently or noncovalently to a macromolecule. While ligands can be naturally present, they can be also artifacts from reagents used for sample preparation or they can be introduced to investigate binding properties. Ligands in a model need to be refined and therefore need restraints. Some ligands are very common, so that geometry restraints are available in dictionaries (Vagin *et al.*, 2004 ▸; N. W. Moriarty & P. D. Adams, http://sourceforge.net/projects/geostd) which can be obtained and updated from a number of sources (Moriarty & Adams, 2019 ▸). Other ligands are rare or novel, requiring that restraints be generated on a case-by-case basis.

*Phenix* has several tools for generating and handling ligand restraints. *eLBOW* (*phenix.elbow*) automatically generates geometry restraints for novel ligands or improves restraints for standard ligands (Moriarty *et al.*, 2009 ▸). *ReadySet* (*phenix.ready_set*) prepares a model for refinement by generating all necessary ligand restraints with *eLBOW* and updating the model file to reflect atom name changes from the new restraints. The tool *REEL* includes a 3D view of the ligand and a tabular view of the restraints, so that target values and standard deviations can be edited easily (Moriarty *et al.*, 2017 ▸).

#### 6.1.3. Other restraints   

Several other types of restraints are available in *Phenix* tools.

(i) Secondary-structure restraints. When data resolution is low, secondary-structure elements (helices and sheets in proteins, base pairs and stacking pairs in nucleic acids) might not correctly maintain their conformation; for example, a helix can lose its regular arrangement. Restraining the hydrogen bonds in the secondary-structure element can help to maintain the regular structure (Headd *et al.*, 2012 ▸).
(ii) Ramachandran-plot restraints. The backbone dihedral angles can be restrained to stay in the allowed regions of the Ramachandran plot (Oldfield, 2001 ▸; Emsley *et al.*, 2010 ▸; Headd *et al.*, 2012 ▸). These restraints can prevent the model from degrading at low resolution when the conformation is approximately correct, but should be used with caution because they can result in an incorrect local conformation minimum when the model geometry is poor (Richardson *et al.*, 2018 ▸).
(iii) Parallelity restraints. Molecules may contain planar atom groups that are approximately parallel to each other, such as base pairs and stacking pairs in nucleic acids. Parallel­ity restraints keep the atom groups parallel (Sobolev *et al.*, 2015 ▸; Richardson, 2015 ▸).
(iv) Rotamer-specific restraints. These restraints lock a particular χ-angle configuration of an amino-acid residue side chain to preserve its valid rotameric state.
(v) NCS restraints. If the asymmetric unit contains two or more similar copies of the same molecule, torsion- or Cartesian-based NCS restraints can be used. NCS-related atoms can be identified automatically or be defined by the user. Torsion-based restraints are generally preferred because they require little or no manual intervention to account for common features such as domains that are very similar in structure but differ in relative orientation (Headd *et al.*, 2014 ▸).
(vi) Reference-model restraints. If the data have low resolution, it can be helpful to use a related structure determined at higher resolution as a reference model to steer refinement (Headd *et al.*, 2012 ▸).

### 6.2. X-ray: *phenix.refine*   

For crystallographic data, refinement is usually performed in reciprocal space, *i.e.* the parameters of the model are changed so that model-derived structure factors match experimental structure-factor amplitudes or intensities. The refinement target in *Phenix* can be expressed either as a least-squares or a maximum-likelihood target. Model parameters, which describe the crystal content and its properties, are a combination of (i) atomic parameters, such as coordinates, ADPs, occupancies and scattering factors, and (ii) non-atomic parameters, which describe contributions arising from bulk solvent, twinning and crystal anisotropy.

*phenix.refine* performs crystallographic structure refinement of atomic and non-atomic model parameters against experimental data (Afonine *et al.*, 2012 ▸) at low to ultrahigh resolutions. Each refinement run begins with bulk-solvent correction and anisotropic scaling (Afonine *et al.*, 2005 ▸, 2013 ▸). The subsequent refinement strategy can be adapted to the data resolution. Useful strategies at low resolution (or in the initial stages) are rigid-body refinement (Afonine *et al.*, 2009 ▸), simulated-annealing refinement in Cartesian or torsion-angle space (Grosse-Kunstleve *et al.*, 2009 ▸) and the detection and use of NCS (Kleywegt & Jones, 1995 ▸). ADP parameterizations include the translation/libration/screw (TLS) model for the movement of groups treated as rigid (Schomaker & Trueblood, 1968 ▸; Urzhumtsev *et al.*, 2016 ▸; Afonine, Adams *et al.*, 2018 ▸) as well as individual isotropic, anisotropic and grouped isotropic ADPs. At ultrahigh resolution (better than 0.7 Å), the interatomic scatterer model can account for residual density from bonding effects (Afonine *et al.*, 2007 ▸). The program also offers occupancy refinement for any user-defined atoms. Water molecules can be placed and updated automatically, and improbable side-chain rotamers can be replaced. In the later stages of refinement it is worthwhile adding H atoms, since they participate in most intermolecular and intramolecular contacts and their presence enables the identification of steric clashes and hydrogen bonds. H atoms can be added at nuclear positions or at electron-cloud center positions (Deis *et al.*, 2013 ▸).

*phenix.refine* is designed to be flexible so that multiple refinement strategies can be combined with each other and applied to any selected part of the model in a single run. As there are several hundred parameters, the protocols can be customized for specific needs. The *phenix.refine* graphical user interface (GUI) is integrated with *Coot* (Emsley *et al.*, 2010 ▸) and *PyMOL* (DeLano, 2002 ▸), so that refined models and associated maps can be readily displayed and analyzed.

While *phenix.refine* is the main crystallographic refinement program of *Phenix*, the following integrated alternatives exist.

(i) A recent addition integrates the Amber molecular-mechanics force field (Case *et al.*, 2018 ▸) for restraints with the functionality of *phenix.refine*. Amber uses energy-based geometry terms and adds electrostatics and van der Waals attractive/dispersive interactions. *Amber* refinement in *Phenix* has been shown to improve model quality, especially sterics and hydrogen bonding at lower resolutions, and to reduce overfitting (Moriarty *et al.*, article in preparation).
(ii) *Ensemble refinement* (*phenix.ensemble_refinement*) combines crystallographic refinement with molecular dynamics to produce ensemble models fitted to diffraction data (Burnley *et al.*, 2012 ▸). The ensemble models can contain ∼50–500 individual copies and can simultaneously account for anisotropic and anharmonic distributions.
(iii) *DEN refinement* (DEN, deformable elastic network) uses a restraint network to maintain local model geometry while allowing larger global domain motions over the course of several cycles of simulated annealing. The protocol is particularly useful for low-resolution diffraction data (Brunger *et al.*, 2012 ▸).
(iv) *Rosetta refinement* (*phenix.rosetta_refine*) integrates the *Rosetta* method for conformational sampling (DiMaio *et al.*, 2013 ▸) with the X-ray targets, ADP refinement and map generation in *phenix.refine*. This tool is useful at low resolution, where it combines a wide radius of convergence across distinct local minima with realistic geometry. It can also be used to prepare crystal structures for further modeling in *Rosetta*.

### 6.3. Cryo-EM: *phenix.real_space_refine*   

The outcome of the single-particle cryo-EM reconstruction is a three-dimensional map, so it is natural to perform refinement of the model in real space. Phases are experimentally determined and are not improved by the procedure.

The recently developed tool *phenix.real_space_refine* was specifically designed to perform refinements in real space (Afonine, Poon *et al.*, 2018 ▸). The algorithm uses a simplified refinement target function that makes calculations faster, so that optimal data-restraint weights can be identified with little runtime cost. In addition to standard restraints on covalent geometry, *phenix.real_space_refine* makes use of secondary-structure, Ramachandran-plot and rotamer-spacific restraints as well as internal molecular-symmetry constraints. The default mode performs gradient-driven minimization of the entire model, but optimization can also be performed using simulated annealing, morphing (Terwilliger *et al.*, 2013 ▸), rigid-body refinement and systematic side-chain improvement (Oldfield, 2001 ▸). As is the case for reciprocal-space refinement, real-space refinement should be alternated with validation and manual corrections. The real-space refinement procedure is robust and works at resolutions from 1 to 6 Å.

### 6.4. Tools for neutron crystallography   

#### 6.4.1. Adding H/D atoms   

The crystals used for neutron diffraction experiments contain H, D or both H and D atoms. If both isotopes are present, some sites (labile or exchangeable sites) can be shared by H and D, *i.e.* some molecules have D at a particular site, while others have H.

*ReadySet* (*phenix.ready_set*) adds H or D atoms to a model file using the *Reduce* algorithm (Word, Lovell, Richardson *et al.*, 1999 ▸). In particular, the tool can add H/D atoms at exchangeable sites of protein amino acids and H or D to water-molecule O atoms. At labile sites, H atoms are placed in alternative location ‘A’ and the corresponding D atoms are placed in location ‘B’.

#### 6.4.2. Joint refinement in *phenix.refine*   

Owing to fairly prohibitive experimental demands, neutron diffraction data from macromolecules typically have low data completeness and a low signal-to-noise ratio. Furthermore, the model contains H (or D) atoms as independent parameters, increasing the number of variables significantly. As an X-ray structure is usually available before a neutron experiment is conducted, it is possible to refine a single model of a macromolecule simultaneously against X-ray and neutron data. This strategy, called joint X-ray and neutron refinement (joint XN refinement), ameliorates the data-to-parameters ratio by increasing the amount of experimental data used in refinement, leading to more complete and accurate models (Coppens, 1967 ▸; Orpen *et al.*, 1978 ▸; Wlodawer, 1980 ▸; Wlodawer & Hendrickson, 1982 ▸).

Macromolecular models can be refined with *phenix.refine* using neutron data or X-ray and neutron data simultaneously (Adams, Mustyakimov *et al.*, 2009 ▸; Afonine *et al.*, 2010 ▸). The program automatically detects exchangeable H/D sites in the model and ensures that the sum of occupancies is equal to one. The position of H (or D) atoms can be refined with a ‘riding model’ (Busing & Levy, 1964 ▸; Sheldrick & Schneider, 1997 ▸) or individually. All standard tools available for X-ray refinement (Section 6.2) are also available for refinement using neutron data.

## 7. Validation   

Validation indicates good parts and highlights problems in macromolecular models, and should guide corrections throughout the structure-solution process. In particular, the refinement stage benefits from validation: the process consists of cycles of validation, rebuilding (either manual or automated) and automated refinement, which are repeated until a satisfactory model is obtained. As problems are corrected, the model quality, refinement behavior and even the density map quality (for X-ray and neutron) all improve.

Validation addresses data, model and model-versus-data quality. This section covers model and model-versus-data quality, as data quality has already been described in Section 3. There are many well established metrics. with new ones being developed to cover emerging needs. Some metrics are global (such as *R*_free_), while others are local (such as a Ramachandran outliers), but each local measure is usually also collected into a global score (such as the clashscore; Word, Lovell, LaBean *et al.*, 1999 ▸). The most diagnostic and reliable validation criteria are those not used in the refinement target, providing independent direction for rebuilding.

In *Phenix*, validation of crystallographic or cryo-EM data and models is performed with the respective *Comprehensive validation* GUIs or on the command line. The underlying principles behind model validation are the same for any experimental method. A good model should make chemical sense and be consistent with empirical statistics for high-quality prior structures. The most useful validation criteria depend on the resolution of the data. Model-versus-data validation depends on the type of experimental data and requires that the model explains its own data well. Generally, the goal is not zero outliers, but as few outliers as feasible (Richardson, Williams, Hintze *et al.*, 2018 ▸). Ideally, each outlier should be explainable by reference to its environment (for example hydrogen bonding and/or steric packing stabil­izing a rotamer outlier) and/or by the experimental data.

### 7.1. Model validation   

In *Phenix*, model validation is provided in the *Comprehensive validation* GUI. Overall model statistics are presented in a summary chart with local scores as graphic plots and as tables that list the outliers on each criterion. Model-validation tasks are essentially identical to the *MolProbity* web service (http://molprobity.biochem.duke.edu/; Chen *et al.*, 2010 ▸; Richardson, Williams, Hintze *et al.*, 2018 ▸; Williams, Headd *et al.*, 2018 ▸).

The *Comprehensive validation* tool uses bond-length and angle target values for proteins, nucleic acids and ligands from the same libraries as are applied for refinement restraints (Section 6.1). Conformational, steric and some special-purpose metrics use the algorithms developed for *MolProbity* (Williams, Headd *et al.*, 2018 ▸) and implemented in *cctbx* (Section 9). C^β^ deviations diagnose side chain–backbone incompatibility around the C^α^ tetrahedron (Lovell *et al.*, 2003 ▸) except when covalent geometry restraints need to be so tight at low resolution that a C^β^ atom cannot deviate from ideal even if its position is incorrect.

**Figure 8:**
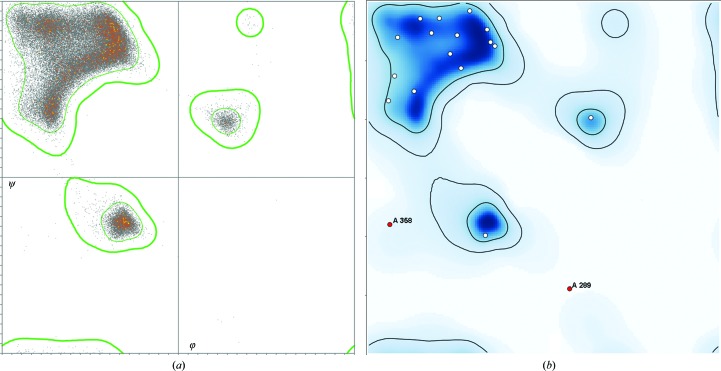
Ramachandran φ, ψ plots for the pre-Pro case. (*a*) The reference distribution of 60 000 well determined pre-Pro residues, with contours that enclose the favored 98% of the data (thin green lines) and that exclude the outliers (thick green lines). (*b*) A pre-Pro Ramachandran plot in the *Phenix* GUI for a query structure, showing two labeled outliers in red. Note that pre-Pro is very different from a general case Ramachandran plot.

Conformational validation relies on the smoothed, multi-dimensional distributions for dihedral-angle combinations from quality-filtered reference data in *MolProbity* (Chen *et al.*, 2010 ▸; Williams, Headd *et al.*, 2018 ▸). Ramachandran backbone scores use six φ, ψ distributions that have quite different outlier contours: general, Ile/Val, Gly, pre-Pro, *trans*-Pro and *cis*-Pro (Read *et al.*, 2011 ▸). Fig. 8 ▸(*a*) shows the underlying pre-Pro data distribution and Fig. 8 ▸(*b*) shows the pre-Pro plot for a query model as shown in the validation GUI. Side-chain rotamer distributions have recently been updated (Hintze *et al.*, 2016 ▸). Omega distributions flag *cis* or twisted peptides. RNA ribose pucker outliers are diagnosed by a simple relationship between the well fit 3′ phosphate and glycosidic bond direction (Richardson *et al.*, 2008 ▸), which also enables pucker-specific geometry targets in refinement (Adams *et al.*, 2010 ▸).

Steric validation is accomplished by adding and optimizing H atoms with *Reduce* and calculating their all-atom contacts with *Probe* (Word, Lovell, Richardson *et al.*, 1999 ▸; Word, Lovell, LaBean *et al.*, 1999 ▸). An overall measure, called clashscore, is the number of serious clashes (non-hydrogen-bond overlap ≥ 0.4 Å) per 1000 atoms. *Reduce* can also correct Asn/Gln/His ‘flips’ and suggest His protonation. Clashes flag problems at any resolution if they occur, but it is possible that the clashscore can be artificially reduced owing to tight nonbonded distance restraints. At high resolution, clashes flag incompatibilities within each alternate conformation model or in disordered regions where geometry and steric restraints have been downweighted or removed.

The *CaBLAM* analysis (Williams, Headd *et al.*, 2018 ▸) was recently developed to validate protein backbone conformations in models determined at low resolution (2.5–4 Å), where it is difficult to determine peptide orientations as carbonyl O atoms cannot be discerned in density maps (this applies for crystallography and for cryo-EM). *CaBLAM* uses virtual C^α^ dihedrals to determine the local chain trace along with a virtual CO dihedral to diagnose where a peptide orientation is incompatible with it. *CaBLAM* outliers are not subject to overfitting, so they provide a less biased quality indicator. Most but not all *CaBLAM* outliers at the 1% level flag a real problem, which usually requires changing the peptide orientation considerably rather than tweaking it across a contour boundary. They can often be corrected manually by modifying peptide orientations or by regularizing the local secondary structure.

Several rare but serious problems are now flagged if they occur, such as *cis*-non-Pro peptides, which are genuine (and typically have a clear structural role) for only one in 3000 residues and have been grossly overused, especially at low resolution (Croll, 2015 ▸; Williams, Videau *et al.*, 2018 ▸). *Cis*-non-Pro peptides cannot be justified by experimental data at resolutions lower than 2.5 Å and should be modeled only if known from other resources (Richardson, Williams, Videau *et al.*, 2018 ▸). H and D atoms are now analyzed if they are present, summarizing relevant properties and flagging issues with H, D or exchanged sites, such as missing atoms, unusual geometry and unlikely occupancies. This is of particular use for models determined by neutron diffraction (Liebschner *et al.*, 2018 ▸).

**Figure 9:**
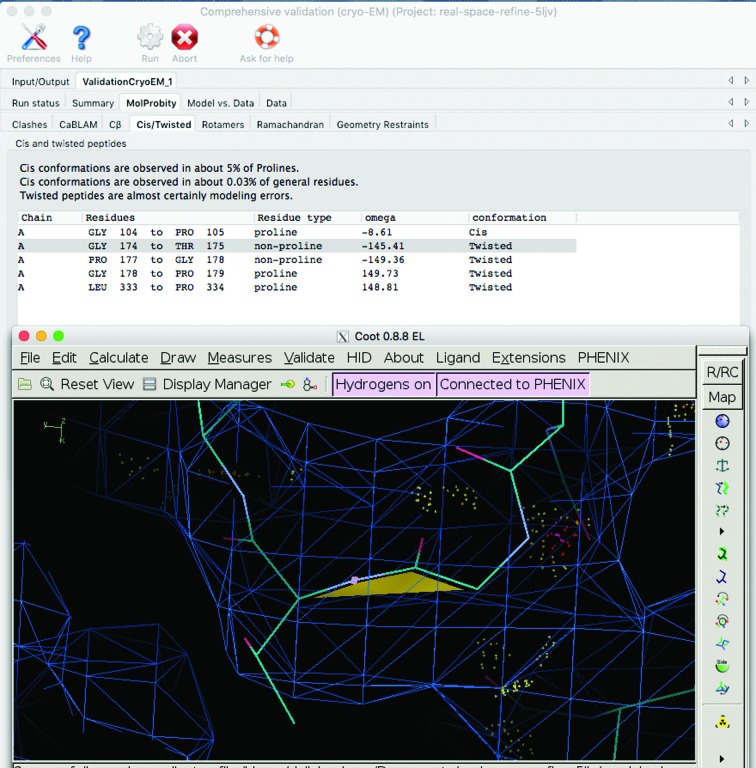
Screenshots of the cryo-EM *Comprehensive validation* tool and a *Coot* window. Clicking on the item in the table of *cis*/twisted peptides (highlighted in gray) recenters the *Coot* window on that peptide.

In the *Phenix* GUI, the results from all specific validations are seamlessly integrated with the graphics programs *Coot* and *PyMOL* (Fig. 9 ▸). Outliers of any type are listed as a table, where clicking on an outlier will recenter the graphics window on that atom or residue. If experimental data were supplied, maps will be displayed as well. The *KiNG* Java-based viewer (Chen *et al.*, 2009 ▸) set up by *phenix.kinemage* displays all model-validation outliers in 3D to highlight local clusters of outliers around single serious problems. Generally, the integration of validation results with graphics programs reduces the effort that is required to fix problems. An extensive guide to the interpretation and use of model validation is available from the *Proceedings of the 2017 CCP4 Study Weekend* (Richardson, Williams, Hintze *et al.*, 2018 ▸).

### 7.2. Model versus data validation   

#### 7.2.1. *Comprehensive validation*: crystallography   

The overall agreement between the model and the diffraction data is measured by *R* factors, which evaluate the difference between the observed (*F*_obs_) and calculated (*F*_model_) structure-factor amplitudes,
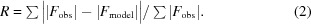



The *R*_work_ value is calculated on the large subset of the diffraction data used for refinement. For cross-validation, *R*_free_ is calculated on a subset of the data (typically about 2000 reflections) that are not used in refinement (Brünger, 1992 ▸). If *R*_free_ increases while *R*_work_ decreases, the model might be incorrectly parameterized or the refinement strategy needs to be revised (Kleywegt & Brünger, 1996 ▸).

*phenix.refine* reports *R*_work_ and *R*_free_ values after refinement; *R*-factor plots show how the values change during the refinement run. The distribution of *R* factors across resolution shells can be used to pinpoint anomalies such as ice rings, saturated reflections or problems with bulk-solvent modeling.

While *R* factors are calculated in reciprocal space, real-space correlation coefficients measure how the model fits the density map locally, for example at the chain, residue or atom level. In *Phenix* validation, residues with low real-space correlation coefficients are listed in a table linked to graphics programs, allowing recentering on the residue in question to enable analysis and correction. This is useful because the correlation may be sometimes misleading.

The *Polygon* tool combines six diverse measures (average ADP, r.m.s.d. bonds and angles, clashscore, *R*_work_ and *R*_free_) to visualize the refinement outcome in radial, one-dimensional histograms (Urzhumtseva *et al.*, 2009 ▸). Lines connecting the scores form a hexagonal polygon that should be small and approximately symmetric. This is similar to the wwPDB slider graphic (https://www.wwpdb.org/validation/2017/XrayValidationReportHelp#overall_quality) that conveys model quality at a glance.

#### 7.2.2. *Comprehensive validation*: cryo-EM   

While validation methods in crystallography have had decades to mature, cryo-EM has only recently evolved into a routine technique for near-atomic resolution models (Kühlbrandt, 2014 ▸). The search for appropriate metrics to assess model and model-to-data fit is therefore still ongoing (Afonine, Klaholz *et al.*, 2018 ▸). One measure of agreement between a model and a map is the model–map correlation coefficient (map CC; Brändén & Jones, 1990 ▸; Jones *et al.*, 1991 ▸). In reciprocal space, model-versus-data agreement is assessed by curves of the Fourier shell correlation (FSC) as a function of resolution. The map–model FSC is the correlation between the Fourier coefficients computed by Fourier transformation of the 3D reconstruction and of a model-based map (Rosenthal & Henderson, 2003 ▸).

The *Phenix* *Comprehensive validation* tool (Fig. 9 ▸) for cryo-EM reports newly developed model and map-to-model quality indicators (Afonine, Klaholz *et al.*, 2018 ▸), such as *CaBLAM* outliers, the CC using the entire map (CC_box_), the CC within a mask (CC_mask_) and the map–model FSC curve. Plots of average CC values per chain and per residue help to identify problematic regions of the macromolecule.

The *EMRinger* (*phenix.emringer*) score quantifies how well the model backbone places side chains in density peaks that are consistent with rotameric conformations (Barad *et al.*, 2015 ▸).

## 8. Other tools   

### 8.1. X-ray   

#### 8.1.1. Electron-density maps   

Electron-density maps are routinely used to guide manual model building of crystallo­graphic structures. Below is a selection of tools for map calculations in *Phenix*. The *Phenix* documentation includes a more complete list (http://www.phenix-online.org/documentation/).

(i) *Polder maps* (*phenix.polder*) uncover weak difference densities by locally excluding bulk solvent (Liebschner *et al.*, 2017 ▸). They are useful for ligands and residues protruding into the solvent area.
(ii) *Composite OMIT maps* (*phenix.composite_omit_maps*) are generated by combining OMIT maps of specific regions to obtain a map covering the entire contents of the unit cell (Brünger *et al.*, 1998 ▸). This map is relatively bias-free without severely compromising phase quality.
(iii) *Feature-enhanced maps* (FEM; *phenix.feature_enhanced_map*) modify a 2*mF*_obs_ − *DF*_model_ σ_A_-weighted map so that weak signals are strengthened while model bias and noise are reduced (Afonine *et al.*, 2015 ▸).

New metrics to compare crystallographic contour maps are available in the *Map Sigma Level Comparison* tool (*phenix.map_comparison*; Urzhumtsev *et al.*, 2014 ▸).

#### 8.1.2. Structure comparison   

It is common to study near-identical protein structures, such as mutants, proteins with different ligands or NCS-related copies. Often, it is useful to compare the structures to find differences and similarities.

The *Structure comparison* tool (Moriarty *et al.*, 2018 ▸) valid­ates and analyzes similar protein models (>80% sequence identity). The GUI displays validation outliers and conformational differences between chains in a table, linked to graphics windows (*Coot* and *PyMOL*). Analyses include ligands, persistent ions and water molecules, rotamers, Ramachandran angles, missing atoms, secondary structure, water locations, ω angles and ADPs. The extracted chains and electron-density maps can be superimposed onto a common frame of reference. The chains may subsequently be edited in *Coot* to ensure consistency and/or fix errors, and then recovered in their original orientations for further refinement and rebuilding.

#### 8.1.3. Ligand fitting   

The goal of a crystallographic study is often to understand the interaction of a small-molecule ligand with a macromolecule. It is also common to discover density for an unanticipated small molecule. In both cases, it is necessary to fit the ligand into the electron density to complete the atomic model.

*Phenix* has several tools to investigate ligands.

(i) *LigandFit* (*phenix.ligand_fit*) identifies difference density peaks in a map and tries to place a user-defined ligand in the density (Terwilliger *et al.*, 2006 ▸, 2007 ▸).
(ii) *Guided ligand replacement* (*phenix.guided_ligand_replacement*) uses prior knowledge about ligand binding in a protein to assist the fitting of a similar ligand into the same or a similar protein (Klei *et al.*, 2014 ▸). This tool helps study a series of compounds for the same or related macromolecular targets.
(iii) *Ligand identification* (*phenix.ligand_identification*) analyzes difference density peaks to reveal which ligand is likely to be present (Terwilliger *et al.*, 2006 ▸, 2007 ▸). The tool uses a library of 180 most frequently observed ligands in the PDB and ranks each molecule by density fit and chemical interactions with the macromolecule.

### 8.2. Using other programs within *Phenix*   

Several programs from external developers can be executed in *Phenix*. Most require separate installation.

(i) *MR-Rosetta* (*phenix.mr_rosetta*) uses homology modeling in the *Rosetta* program to improve a model before and/or after MR (DiMaio *et al.*, 2011 ▸; Terwilliger *et al.*, 2012 ▸). Once a potential solution is obtained, *Rosetta* fills in missing sections and rebuilds the model to improve the fit to the electron-density map and the phases for map interpretation or automated model building. This approach is helpful in cases where the MR model differs greatly from the target.
(ii) *ERRASER* (Chou *et al.*, 2013 ▸) improves RNA backbone conformations by combining *MolProbity* clash analysis, *Phenix* refinement and a pruned enumeration and optimization in *Rosetta*.
(iii) Conventional restraints may not capture the influences of intermolecular covalent and nonbonded interactions, metal coordination or solvation. The semiempirical quantum mechanics engine *DivCon* has been integrated into *phenix.refine* to create gradients for a region of interest (Borbulevych *et al.*, 2014 ▸).
(iv) *CryoFit* uses molecular dynamics to perform flexible fitting of a model to a cryo-EM map (Kirmizialtin *et al.*, 2015 ▸; Kim *et al.*, 2019 ▸). The approach produces a new conformational model with optimized atomic coordinates and preserved stereochemistry and secondary structure.
(v) Quantum refinement (*Q*|*R*) is a method to refine macromolecular models with restraints derived from quantum chemistry instead of library-based restraints (Zheng, Moriarty *et al.*, 2017 ▸; Zheng, Reimers *et al.*, 2017 ▸). The *Q*|*R* source code (https://github.com/qrefine/qr-core) uses *cctbx* (Section 9.1) to construct a refinement protocol resembling *phenix.refine*.
(vi) *ISOLDE* (Croll, 2018 ▸) is a plugin to *UCSF ChimeraX* (Goddard *et al.*, 2018 ▸) for improving low-resolution cryo-EM or crystal structures. It performs interactively guided simulation with molecular-dynamics flexible fitting against a map and real-time validation; it will soon be possible to launch it from the *Phenix* GUI.
(vii) Cryo-EM model building with *Pathwalker* (Baker *et al.*, 2012 ▸; Chen *et al.*, 2016 ▸) will soon be available within *Phenix*. *Pathwalker* can construct protein backbone models directly from near-atomic resolution cryo-EM density maps using a modified approach to traveling salesman problem solvers. When coupled with *Phenix* tools, such as *phenix.pulchra* (Rotkiewicz & Skolnick, 2008 ▸) and real-space refinement, complete atomistic models can be generated within a few minutes for individual proteins or complexes.

### 8.3. Tools for model deposition   

Several tools are available to facilitate the deposition process of macromolecular structures.

(i) *Generate Table 1 for journal* (*phenix.table_one*) is a tool for generating the standard table of crystallographic statistics required by most scientific journals for structure solutions. It summarizes the validation statistics and calculates merging statistics for crystallographic data. For cryo-EM structures, the *Comprehensive validation* (CryoEM) GUI has a button to generate a similar table containing the model-validation statistics as well as map resolution estimates.
(ii) *Prepare model for PDB deposition* (*mmtbx.prepare_pdb_deposition*) adds the sequence information to the model file. In particular, the tool creates model files in PDBx/mmCIF format, which have been mandatory for crystallographic depositions to the PDB since 1 July 2019 (Adams *et al.*, 2019 ▸).
(iii) *Get PDB validation report* (*phenix.get_pdb_validation_report*) retrieves the validation report from the wwPDB through their web interface. By providing the model and optional data in mmCIF format, the validation report can help users to identify problems before starting the deposition process.

## 9. Infrastructure   

### 9.1. Architecture   

*Phenix* is built on the *Computational Crystallography Toolbox* (*cctbx*), which is an open-source library (https://github.com/cctbx/cctbx_project) of reusable software components for macromolecular structure determination (Grosse-Kunstleve *et al.*, 2002 ▸). The *cctbx* components are written both in a compiled language (C++) and a flexible scripting language (Python; Lutz & Ascher, 1999 ▸). This approach is very efficient because high-level algorithms (such as refinement protocols) can be rapidly developed in the scripting language, while computationally intensive algorithms can be implemented in the compiled language. The Boost.Python Library (http://www.boost.org/) is used to expose the C++ interfaces, classes and functions to Python (Abrahams & Grosse-Kunstleve, 2003 ▸).

The GUI is scripted through Python and produces a ‘native’ look^5^ on each operating system. The current *Phenix* release (v.1.16) includes GUIs for all major programs. Embedded graphs are computed with the free *matplotlib* Python library (Hunter, 2007 ▸). A simple 3D graphics viewer can be used to display the molecule or to pick atom selections interactively.

### 9.2. Documentation   

**Figure 10:**
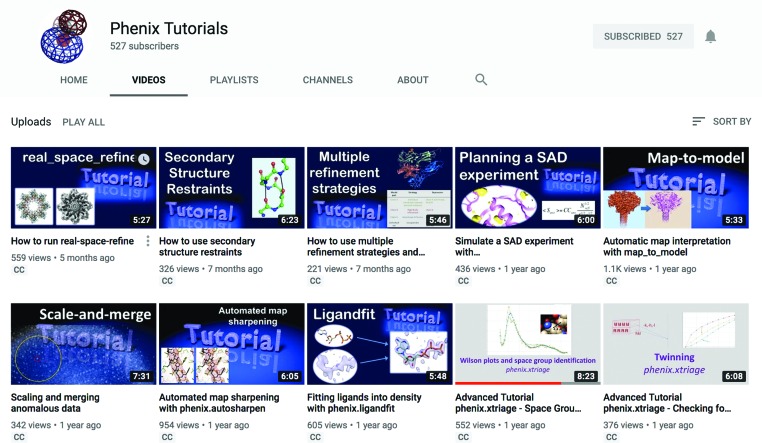
The videos on the *Phenix* tutorials YouTube channel cover the main *Phenix* programs, refinement strategies and lectures.

The *Phenix* GUI provides more than 175 tools, with even more programs available on the command line (∼500). The extensive online manual covers about 180 separate HTML pages, describes GUI and command-line versions of individual programs and includes tutorials and FAQs. The *Phenix* Tutorials YouTube channel (https://www.youtube.com/c/phenixtutorials; Fig. 10 ▸) currently provides 29 tutorial videos. Each video introduces a *Phenix* tool, summarizes the input files and parameters, explains how to run the program and discusses the results.

## 10. Conclusion   

The *Phenix* software for macromolecular structure determination handles data from three experimental methods: cryo-EM, X-ray diffraction and neutron diffraction. All steps in the structure-solution process are addressed by programs that are tailored for the type of experimental data, but share algorithms where appropriate. Procedures are automated to minimize repetitive and time-consuming manual tasks as far as feasible. For the future, the improvement of automated model building, refinement and validation at low resolution (worse than 3 Å) remains a priority; another area of development to help the structural biology community is the automated identification, fitting and refinement of ligands, ions and water.

Many challenging opportunities still exist in crystallography and cryo-EM owing to advances in light sources and instrumentation that make it possible to go beyond structure determination by single-crystal diffraction and single-particle cryo-EM. For example, free-electron lasers (FELs) and serial synchrotron crystallography (Chapman *et al.*, 2011 ▸; Rossmann, 2014 ▸; Diederichs & Wang, 2017 ▸; Standfuss & Spence, 2017 ▸; Schlichting, 2015 ▸) have opened up new approaches to studying the dynamics of macromolecules. Therefore, methods are needed to extract models of molecular motion from time-resolved diffraction experiments. Diffuse X-ray scattering (Wall *et al.*, 2014 ▸, 2018 ▸) can also reveal molecular motions and lattice disorder, but methods to exploit the information contained in diffuse scattering are still scarce. Micro-electron diffraction (MicroED; Liu *et al.*, 2017 ▸; Gruene *et al.*, 2018 ▸; Jones *et al.*, 2018 ▸; Nannenga & Gonen, 2018 ▸) is an emerging technique to determine high-resolution structures of macromolecules. Current procedures for diffraction data will require fine-tuning to treat MicroED data adequately. Similarly, while cryo-EM is typically currently applied to large molecules and complexes, it is becoming increasingly possible to look at smaller molecules because of improvements in instrumentation and data processing. Furthermore, the use of focused refinement of cryo-EM data (von Loeffelholz *et al.*, 2017 ▸; Natchiar *et al.*, 2017 ▸) can generate much improved local reconstructions, but it remains to be seen how these can be best combined for model generation and subsequent model refinement. Finally, cryo-tomography, which is a type of electron microscopy that can probe entire cells and thus enable the visualization of molecules *in situ*, nowadays produces reconstructions at better than 10 Å resolution and in some cases significantly better. There will be an increasing need to accurately and effectively combine such lower resolution information with results from high-resolution crystallographic or cryo-EM experiments.

## Figures and Tables

**Figure 3:**
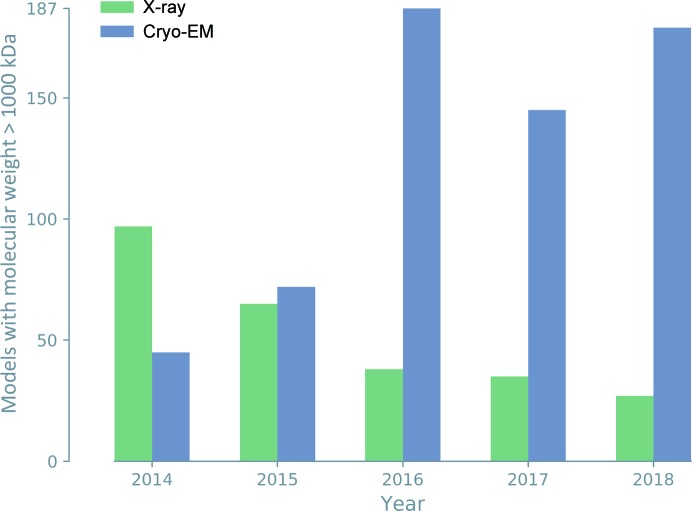
Since 2015, cryo-EM depositions have accounted for the majority of large macromolecular structures.

**Figure 6:**
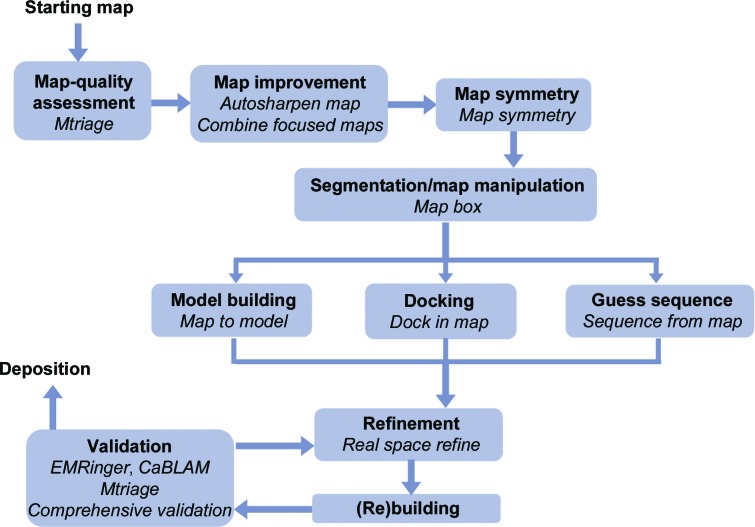
The primary tools for cryo-EM in *Phenix*.

## Footnotes

1 A consensus for the name is not yet established: both the terms ‘cryo-electron microscopy’ and ‘electron cryo-microscopy’ can be found in the literature (Jensen, 2010 ▸).

2 The number of negative intensities after detwinning as a function of the twin fraction.

3 MAD, multiple-wavelength anomalous diffraction; MIR, multiple isomorphous replacement; SIR, single isomorphous replacement.

4 Exceptions exist: for example, some procedures fill in missing reflections with hypothetically derived values (Sheldrick, 2008 ▸).

5 The application takes on the convention, gestures and esthetics of their operating system.
